# Parents’ Willingness to Invest in Primary Oral Health Prevention for Their Preschool Children

**DOI:** 10.3390/ijerph182111437

**Published:** 2021-10-30

**Authors:** Peggy C.J.M. van Spreuwel, Katarina Jerković-Ćosić, Cor van Loveren, Geert J.M.G. van der Heijden

**Affiliations:** 1 Research Group Innovation in Preventive Care, Utrecht University of Applied Science (HU), 3584 Utrecht, The Netherlands; katarina.jerkovic@hu.nl; 2 Department of Oral Public Health, Academic Centre for Dentistry Amsterdam (ACTA), University of Amsterdam and Vrije Universiteit Amsterdam, 1081 Amsterdam, The Netherlands; geert.vander.heijden@acta.nl; 3 Department of Preventive Dentistry, Academic Centre for Dentistry Amsterdam (ACTA), University of Amsterdam and Vrije Universiteit Amsterdam, 1081 Amsterdam, The Netherlands; c.van.loveren@acta.nl

**Keywords:** willingness to invest, contingent valuation, willingness to pay, preschool children, oral hygiene behaviour, dental caries, preventive dentistry

## Abstract

There is growing evidence for the beneficial effects of starting oral health prevention early in life. Preventing dental caries in very young children requires considerable investment from parents. Therefore, this cross-sectional study aimed to explore parents’ willingness to pay (WTP) and willingness to invest in time (WTIT) for primary oral health prevention in preschool children and describe whether these are related to the parents’ demographic, socio-economic and behavioural characteristics. In a convenience sample of parents of preschool children aged six months to four years (*n* = 142), data were collected with questionnaires. On average, parents were willing to pay EUR15.84 per month, invest time for 1.9 dental visits per year, and spend 2.4 min per day brushing their child’s teeth. A higher education level of the mother and having a child older than two were associated with a higher WTIT in brushing minutes per day (*p* = 0.03). In addition, parents who brushed their child’s teeth more frequently were also more willing to invest in brushing minutes (*p* < 0.01) and money (*p* < 0.01). Findings emphasise the importance of early oral health interventions and the need to increase awareness of primary prevention’s importance in maintaining healthy teeth and reducing possibly oral health inequalities.

## 1. Introduction

Dental caries is still a major problem worldwide [1], though it could be prevented in most cases. A bad start with oral health maintenance in early childhood may result in caries in the primary teeth, which often predicts caries in permanent dentition [2,3,4]. Young children depend on their parents for their health behaviours, daily hygiene, lifestyle habits and oral health maintenance, including tooth brushing, eating behaviours, and dental visits. Preventing dental caries, especially in preschool children, requires considerable investment from parents. Therefore, it involves a trade-off in investing resources, notably money and time, against the anticipated benefits. To date, parents’ willingness to invest in resources to prevent dental caries in their offspring is rarely studied. 

Assessment of the willingness-to-pay (WTP) can be used for eliciting valuations of non-market goods such as health benefits. The WTP refers to the maximum amount of money that an individual is hypothetically willing to invest in obtaining the benefits of a health program in terms of improved health [5]. An increase in WTP means that people are willing to make a greater offer and prefer the intervention or treatment. It is well described that differences in the ability to pay may translate into differences in WTP [5,6,7]. However, in the Netherlands, WTP for oral health prevention in children is hypothetical because parents’ compulsory health insurance fully covers oral healthcare costs for children up to eighteen.

Regarding preventive dentistry, especially in preschool children, parents’ willingness to invest in time (WTIT) can be considered equally important as WTP [7]. A distinction can be made between WTIT for self-care (e.g., time for tooth brushing) and WTIT for professional care (e.g., time for dental visits). Caries is preventable with good self-care; therefore, the WTIT might be even more important than WTP.

Information about the value parents assign to good oral health for their children, their WTP and WTIT, is relevant for policymakers in designing programs to prevent childhood caries [6,7,8]. Consequently, this may lead to more tailored dental care in individual patients. Despite the increasing popularity of WTP studies in dentistry, only a few studies have assessed parents’ WTP to prevent childhood caries [6,7,8,9,10,11,12,13]. Tianviwat et al. [9] valued the WTP for prevention (sealant program) versus cure (filling program) among Thai parents of primary school children. They found no differences between the two programs, indicating, according to the authors, that parental preferences might be a barrier to the prevention of caries among schoolchildren. Walshaw et al. [10] found that WTP values for Brazilian parents differ from those for UK parents of six to sixteen-year-olds and are therefore specific to the social and cultural circumstances and the individual context, such as behavioural and demographic factors. In addition to WTP, two studies also measured WTIT for brushing minutes per day and dental visits per year. Berendsen et al. [12] found a significantly higher mean number of decayed missing and filled teeth (dmft) in five to six-year-old children whose parents were more willing to pay and visit the dentist more often, while the mean dmft was significantly lower in children of parents who were willing to invest more time in tooth brushing. The latter might suggest that children are better off when parents are willing to invest time for self-care [12]. Vermaire et al. [11] found that 12% of parents were unwilling to pay any money, brush more than two minutes per day, or go to the dentist more than once a year. These results indicate a clear challenge for oral health prevention in children whose parents are unwilling to invest money and time. 

At present, very little is known about WTP and WTIT in parents of preschool children. Only one UK study performed a public preference survey that included the WTP of preschool children’s parents and found that parents’ valuation for dental caries prevention was higher if it concerned permanent teeth compared to primary teeth [13]. With the growing amount and quality of evidence for beneficial effects of starting oral health prevention early in life, preferably around first tooth eruption [14,15,16], it is interesting to investigate WTP and WTIT for primary prevention among parents of preschool children. Therefore, this study aims to explore the WTP and WTIT for primary prevention in parents of preschool children and to describe whether these are related to the parents’ demographic and socio-economic characteristics and behavioural attributes.

## 2. Materials and Methods

### 2.1. Participant Recruitment and Data Collection

For this cross-sectional research, participants were recruited at well-baby clinics (*n* = 4) and swimming pools (*n* = 2) in two large cities (The Hague and Zoetermeer) in the Netherlands. A convenience sampling approach was used to recruit participants. In April 2016, two researchers asked all parents with children aged six months to four years who entered the selected well-baby clinic or swimming pool to participate in this study on different days and times. Participation involved completing a 24-item paper-based questionnaire, which would take about ten minutes. The questionnaire included three sections:

1. Demographic variables (7 items);
2. Perceptions, knowledge, and behaviour on the oral health of children (14 items);
3. Willingness to invest money and time in their child’s oral health (3 items).

After oral informed consent from participants for their voluntary participation, they were asked to return a completed questionnaire. Parents who did not complete the questionnaire between entry and leaving the well-baby clinic or swimming pool were considered non-response. 

Section 1—demographic variables

Participants were asked seven questions on demographic topics, including their relationship to the child (‘mother’, ‘father’ or ‘other primary caregiver/guardian’); family composition (living with or without a partner); the age of the considered child; the total number of children within the family and rank of the child concerned (i.e., first child or subsequent). Cultural belonging was asked with the question: ‘To which cultural group do you belong most?’ followed by a list of cultural groups and options to complete. We used the highest completed level of education of the mother to measure socio-economic position and categorised this according to the International Standard Classification of Education (ISCED) as low (no or primary education and lower secondary education, ISCED 0–2), middle (upper secondary education, ISCED 3–4) and high (tertiary education (ISCED 5–8) education.

Section 2—Perceptions, knowledge, and oral health behaviour

Participants were asked to give their assessments on the importance of general health and oral health on a numeric rating scale (NRS) (0 = not important at all—10 = very important); their knowledge about oral health (‘sufficient’ or ‘insufficient’) and to rate their perceptions of the child’s dental status compared to peers (‘better’, ‘the same’ or ‘worse’). Questions on oral health behaviour included frequencies of supervised tooth brushing and independent tooth brushing of the child (‘never’, ‘once a day or less’ and ‘twice a day or more’ ); the ease and comfort for the parents to brush the child’s teeth (NRS: 0 = very easy -10 = very difficult); the age at which toothbrushing started (in months); the use of fluoridated toothpaste (‘yes’, ‘no’, ‘I do not know’), the frequency of in-between-meal sugar consumption (‘four times a day or less’ or ‘more than four times a day’); whether the child has already been to the dentist and the age at the first visit (‘yes, at age …year’ or ‘no’) and the reason for the dental visits (‘check-up’, ‘prevention’, ‘pain/treatment’ or ‘other,...’). In addition, the oral hygiene behaviour of the parents’ was questioned in the same way. 

Section 3—Willingness to invest (primary outcome)

Parents’ WTP and WTIT for their child’s oral health was evaluated by using an existing set of questions [11]: “How much...[investment]...would you be willing to spend maximally to keep your child free of pain and dental caries until his or her 18th birthday?”

1. “...euros per month (answer options: ‘0 euro per month’, ‘1–10 euros per month’, ‘11–25 euros per month’, ‘26–50 euros per month’, and ‘more than 50 euros per month’)?”
2. “...number of dental visits per year (answer options: ‘0 visits’, ‘1–2 visits’, ‘3–4 visits’, ‘5–6 visits’ and ‘more than 6 visits’)?”
3. “...toothbrushing minutes per day (answer options: ‘0 min’, ‘1–2 min’, ‘3–4 min’ and ‘more than 4 min’)?”

The standard approach [17], using the midpoint of the payment card interval (e.g., EUR 1–10 has been recoded to EUR 5.5, EUR 11–25 to EUR 18, EUR 26–50 to EUR 38 and more than EUR 50 to EUR 51), has been used to calculate means, standard deviations and a 95% confidence interval for WTP and WTIT outcomes. 

### 2.2. Data Analysis

All data were entered into SPSS (IBM SPSS 25.0, IBM, Armonk, NY, USA). Tables were used to describe and evaluate the value distribution of all variables. Chi-square tests were used to investigate bivariate associations between the categorical dependent variables (primary outcome), notably the parents’ WTP and WTIT, and the categorical independent variables, notably demographic and socio-economic characteristics and oral hygiene behaviours. Complete case analysis was used to handle missing data. A *p*-value of 0.05 or below was considered to indicate a statistically significant association. 

## 3. Results

### 3.1. Study Sample and Response

Of the 200 questionnaires distributed, 142 were completed (response rate 71%). Lack of time was the most frequently cited reason for refusing participation. The children’s mean age was 2.1 (standard deviation (SD): 1.2) years. Most of the questionnaires were completed by the mother (74%) or father (24%) of the child and occasionally (2%) by other guardians. Table 1 presents a complete description of the study sample. 

### 3.2. Perceptions, Knowledge and Oral Health Behaviour

Table 2 describes this study sample’s general and oral health perceptions, knowledge, and behaviours. Overall, parents considered their child’s general and oral health very important (mean score: 9.7; SD 0.7 and 9.5; SD 0.9, respectively). Most parents felt that they had sufficient knowledge of oral health (96%), and 87% indicated no need for extra information or guidance to prevent dental caries in their children. Parents rate their child’s oral health as better (17%) or equal (82%) to peers, and only one parent reported worse than peers. On average, parents started to brush their child’s teeth at the age of 8.9 (SD 4.5) months, and most parents (92%) brush their child’s teeth at least once or twice daily. About two-thirds (62%) of the children had visited a dentist, with a mean age for the first dental visit being 1.6 (SD 0.9) years of age. 

### 3.3. Willingness to Invest in Oral Health

Table 3 shows the responses to the WTP and WTIT questions. Almost all parents were willing to invest at least EUR 1–10 per month, 1–2 dental visits per year, and 1–2 min of toothbrushing per day to keep their child caries- and pain-free until their eighteenth birthday. Only a few parents (5%) said they did not want to invest any money or did not want to invest time in dental visits (1%). The WTP was widely and skewed distributed, ranging from EUR 0 to more than EUR 50 per month with a EUR 1–10 modus per month. On average, parents were willing to pay EUR 15.84 per month (SD EUR 14.40; median EUR 5.50); to invest time for 1.9 dental visits per year (SD 1.1; median 1.5); and 2.4 (SD 1.3; median 2.5) minutes a day on brushing their child’s teeth. 

### 3.4. Willingness to Invest and Demographic Variables 

Table 4 shows the bivariate analysis results between demographic variables and the WTP and WTIT questions. Significant higher WTIT in brushing minutes per day was found in parents with a higher level of education and having children aged two years or older. In the highest education category, 64% were willing to brush for more than 3 min a day compared to 42% in the low to medium educated category (χ^2^ (2) = 6.960, *p* = 0.03). When the child was over two years of age, 63% were willing to brush for at least 3 min compared to 40% for children under two (χ^2^ (2) = 6.982, *p* = 0.03).

### 3.5. Willingness to Invest and Oral Hygiene Behaviour

Table 5 shows the bivariate analysis results between oral hygiene behaviour and the WTP and WTIT questions. The child’s brushing frequency seemed to be positively associated with parental WTP and WTIT in brushing minutes. Parents who brushed their child’s teeth more frequently were more willing to invest in money per month ((χ^2^ (2) = 12.751, *p* = 0.00) and were more willing to invest time in toothbrushing (χ^2^ (2) = 12.729, *p* < 0.01). The same was found for parents’ oral hygiene behaviour and the WTP (χ^2^ (2) = 9.932, *p* = 0.01. 

## 4. Discussion

In this study, we aimed to explore parents’ willingness to invest in primary oral health prevention for children in preschool age; and to describe whether their willingness to invest is related to demographic and socio-economic characteristics and behavioural aspects.

Almost all parents in this study are willing to invest in money and time to maintain good oral health until the child’s eighteenth birthday. Only 5% indicated to be unwilling to pay any money, and 1% was unwilling to invest any time in brushing. Parents who brush their teeth or brush their child’s teeth twice a day are more willing to invest in oral health in money and brushing minutes. The same applies to parents of children over two years of age. We also found a higher WTIT in brushing minutes when mothers had a higher level of education completed.

To the best of the authors’ knowledge, this study is the first on WTP and WTIT for primary oral health prevention in preschool children, which hampers comparison with other studies in the same population. Previous research on this topic by Vermaire et al. and Berendsen et al. focused on willingness to invest among parents of schoolchildren, aged six to nine years old [11] and five to six years old [12] and recruited parents of children enrolled in a clinical trial or through a paediatric dental clinic. Parents in these studies were willing to pay almost the double amount per month, visiting the dentist 1 to 1.5 times more often per year, and brushing 2–4 min longer daily (i.e., EUR 31.25 and EUR 37.00 per month; 3.5 and 3.0 dental visits per year; and 6.5 and 4.5 brushing minutes per day, respectively) compared to parents in our study. Berendsen et al. also showed that parents of children with a lower number of decayed, missing, or filled teeth (dmft) were significantly more willing to spend more time on tooth brushing (mean dmft difference of 1.3 between lowest and highest WTIT categories for toothbrushing). At the same time, parents of children with a higher dmft were significantly more often willing to pay the highest amount of money (mean dmft difference of 1.2 between lowest and highest WTP categories) and visit the dentist more often (mean dmft difference of 1.3 between lowest and highest WTIT categories for dental visits per year) [12]. Oscarson et al. studied nineteen-year-olds and found that respondents with a high caries risk were willing to pay more monthly for preventive care than respondents with a low caries risk (Swedish Krona (SEK) 117.1 and SEK 90.6, respectively) [18]. Other studies have also described this phenomenon that people are more willing to invest in prevention when exposed to the disease or disorder [5,8] and might explain the lower willingness to invest found in our study population.

Dutch people are used to visiting a dentist twice a year. It is common knowledge and a tradition that has been evolved due to transformations in oral health care funding over time. Knowing this may also explain similar results in parents’ willingness to invest in time for dental visits amongst different levels of education, cultures, number of children, and child’s ages, and is in line with Berendsen et al. [12]. In our study, approximately 80% are willing to visit the dentist maximally twice a year for preventive reasons, indicating socially accepted responses. Parents consider their knowledge on oral health and oral health behaviour as sufficient, and they feel no need for further advice or education in this matter, which could also explain the low willingness to visit a dentist more than twice a year. Because most behavioural interventions in (pre)school children require more frequent visits to a dental clinic, more attention should be paid to parents’ awareness of the importance of primary prevention early in life.

As we see in our study, a large group of two and four-year-old children had not visited a dentist yet. Primary care providers at well-baby clinics give some essential advice (e.g., the use of fluoridated toothpaste; first dental visit at the age of two); however, they lack time and attention for oral health prevention [19]. As a result, this group of children does not receive individual primary prevention and have an increased chance to visit a dentist only when caries or pain occurs. A systematic review of Soares et al. [15] and Riggs et al. [16] emphasise the increasing amount of evidence that an early visit to an oral healthcare professional is beneficial for children’s oral health. In January 2021, a renewed clinical practice guideline on preventing dental caries in children was introduced in the Netherlands, requiring primary care providers at well-baby clinics to refer children to oral healthcare professionals around the first tooth’s eruption [20]. However, we argue if this measure alone is sufficient to decrease caries prevalence in Dutch children. Considering that primary care providers at well-baby clinics already advise visiting a dentist around the age of two, only 39% of children were seen in practices by the age of four [21].

### Limitations and Strengths

Interpretation of the findings requires some caution. Firstly, in design, we refrained from hypothesis testing for causality and prior sample size calculation since this study was the first exploration in this population. Moreover, the relatively modest size was too small for a reasonably informative multivariate data analysis, and therefore only univariate and bivariate associations were reported. Secondly, by recruiting parents, we used convenience sampling. Thus, our study participants do not represent a random sample of Dutch parents with preschool children. Parents with a higher level of education represented 62% of our study population, whereas, in the overall Dutch population, the percentage of higher educated persons is around 37% [22]. As an indicator of socio-economic status, the education level has been described frequently as an important factor in oral health behaviour, beliefs, and outcomes [23,24]. Based on previous research, this may have caused a bias towards a possible overestimation in the WTP and the WTIT for brushing minutes [6,11,12]. Thirdly, in the Netherlands, dental care for children up to eighteen years is fully reimbursed within the parents’ compulsory basic healthcare insurance package, which renders the question about WTP for dental care hypothetical. As a result, there may be an increased variation in responses (i.e., protest zero’s) when completing questions on the WTP. Lastly, the set of questions and answer options we used to measure the WTP and WTIT is also known as “the payment card method” and is the most frequently used method to elicit valuations and reflect actual life situations [25]. Nevertheless, it may allow respondents to choose a socially desirable response that may depend on the investment categories presented, resulting in a 10%–30% overestimation of the actual WTP and WTIT [8]. To adjust for this drawback of the payment card method, we used the interval midpoint for the monetary valuation instead of calculating mean WTP values, as Cameron and Huppert advised [17].

Despite the limitations in this study’s design, the results revealed relevant information, indicating that oral health prevention in preschool children is valued lower by parents than prevention in schoolchildren in other studies. The very young age, the low number of teeth erupted, and the ex-ante state (not exposed to the disease yet) may contribute to parents not feeling the urge to invest more in their child’s oral health. From a preventive point of view, it is somewhat worrying that lower educated parents and parents with less favourable oral health behaviour are less willing to invest in their child’s oral health.

## 5. Conclusions

In conclusion, parents in this study are willing to pay a mean of EUR 15.84 per month, invest time for 1.9 dental visits per year, and spend 2.4 min per day brushing their child’s teeth. Furthermore, a higher education level of the mother (*p* = 0.03) and having a child older than two (*p* = 0.03) were associated with a higher willingness to invest in brushing minutes per day. In addition, parents who brushed their child’s teeth more frequently were also more willing to invest in money (*p* < 0.01) and in brushing minutes (*p* < 0.01). These findings emphasise the importance of early oral health interventions and the need to increase awareness of primary prevention’s importance in maintaining healthy teeth and reducing possibly oral health inequalities.

Policymakers can use these findings to efficiently target the population and intervene more upstream with early referral to oral healthcare professionals and interprofessional collaboration with well-baby clinics and other primary care providers [26]. For oral healthcare providers, they should raise awareness of the importance of primary prevention by focusing on parents’ risk perception, outcome expectations and self-efficacy.

## Figures and Tables

**Table 1:** Description of the study sample.

Demographic Variables	*n* (%)	Mean (SD)
Respondent		
Mother	105 (74)	
Father	34 (24)	
Other caregivers	3 (2)	
Cultural belonging		
Western culture	122 (86)	
Non-western culture	20 (14)	
Maternal education level		
Lower education (ISCED 0–2)	17 (12)	
Middle education (ISCED 3–4)	36 (26)	
Higher education (ISCED 5–8)	87 (62)	
Marital status		
Married or Living together	127 (89)	
Single parent	15 (11)	
Number of children		
One child	58 (41)	
Two children	60 (42)	
More than two children	24 (17)	
Child’s age in years		2.1 (1.2)
<1 year old	15 (11)	
1 year old	30 (21)	
2 year old	35 (25)	
3 year old	44 (31)	
4 year old	18 (13)	

**Table 2:** Perceptions, knowledge, and oral hygiene behaviour.

Topics	*n* (%)	Mean (SD)	Median (IQ)
*Perceptions and self-reported oral health knowledge*			
Importance of child’s general health *		9.7 (0.7)	10 (0)
Importance of child’s oral health *		9.5 (0.9)	10 (1)
How easy is it for you to brush your child’s teeth? ^†^		4.7 (3.0)	5 (5)
How do you rate your child’s oral health compared to peers? ^‡^			
Better than peers	24 (17)		
Same as peers	116 (82)		
Worse than peers	1 (1)		
How do you rate your knowledge about oral health?			
Sufficient	137 (96)		
Not sufficient	5 (4)		
Would you prefer extra information or guidance about your child’s oral health			
Yes	18 (13)		
No	124 (87)		
*Oral health behaviour*			
At what age (in months) did you start brushing your child’s teeth? ^§^		8.9 (4.5)	
How often do you (or your partner) brush your child’s teeth?			
Never/occasionally	11 (8)		
one time a day	57 (40)		
≥two times day	74 (52)		
How often does your child brush his or her teeth independently?			
Never/occasionally	87 (61)		
≤one time a day	35 (25)		
≥two times day	20 (14)		
Do you use fluoridated toothpaste for your child?			
Yes	68 (48)		
No	26 (18)		
Don’t know	44 (31)		
My child does not use toothpaste yet	4 (3)		
How many in-betweens (drinks and food) does your child usually have?			
*(water or tea without milk and/or sugar excluded)*			
≤four times a day	109 (71)		
>four times a day	45 (29)		
Have you already been to the dentist with your child?			
Yes	89 (57.4)		
No	66 (42.6)		
Did you already visit the dentist with your child (specified to age)?			
*(% presents the children that visit the dentist in that particular age group)*			
0–1 years old (*n* = 45)	5 (11)		
2 years old *n* = 35)	21 (60)		
3 years old (*n* = 44)	44 (100)		
4 years old (*n* = 18)	16 (89)		
Age (in years) at the first dental visit		1.6 (0.9)	2.0 (4.0)
What is usually the reason for a dental visit with your child? (*n* = 89)			
Preventive check-up	88 (98.9)		
Treatment	1 (1.1)		
How often do you brush your teeth?			
Never	1 (0.6)		
≤one time a day	29 (18.7)		
≥two times a day	125 (80.6)		
How many in-betweens (drinks and food) do you usually have?			
*(water or tea/coffee without milk and/or sugar excluded)*			
≤four times a day	68 (47.9)		
>four times a day	74 (52.1)		

* Rated on a scale ranging from 0 (not at all important) to 10 (very important). ^†^ Rated on a scale ranging from 0 (Very easy) to 10 (very difficult). ^‡^ Missing: *n* = 1; ^§^ Missing: *n* = 4.

**Table 3:** Willingness to invest in child’s oral health.

Willingness to Invest	*n* (%)	Interval Midpoint WTP and WTIT	
Willingness to pay			
EUR 0 per month	7 (5)	Mean (SD)	15.8 (14.4)
EUR 1–10 per month	67 (47)	95% CI	13.5–18.2
EUR 11–25 per month	41 (29)	Median (IQ)	5.5 (12.5)
EUR 26–50 per month	18 (13)		
More than EUR 51 per month	9 (6)		
Willingness to visit the dentist			
0 visits per year	-	Mean (SD)	1.9 (1.1)
1–2 visits per year	121 (85)	95% CI	2.7–2.1
3–4 visits per year	15 (16)	Median (IQ)	1.5 (0.0)
5–6 visits per year	1 (1)		
More than 6 visits per year	5 (4)		
Willingness to brush			
0 min per day	1 (1)	Mean (SD)	2.4 (1.3)
1–2 min per day	62 (44)	95% CI	2.2–2.6
3–4 min per day	66 (47)	Median (IQ)	2.5 (2.0)
More than 4 min per day	13 (9)		

**Table 4:** Distribution (%) of socio-demographic variables by the willingness to invest in children’s oral health.

		Willingness to Pay (Euro per Month)				Willingness to Visit the Dentist (Times per Year)			Willingness to Brush (Minutes per Day)			
	*n*	0–10 (%)	11–25 (%)	≥26 (%)	*p* *	0–2 (%)	≥3 (%)	*p* *	0–2 (%)	3–4 (%)	≥5 (%)	*p* *
Education level (mother)					0.32			0.16				0.03
High	87	47	32	21		88	12		36	54	10	
Low–middle	55	60	24	16		80	20		58	35	7	
Cultural belonging					0.45			0.48				0.73
Western culture	122	50	30	20		86	14		43	47	10	
Non-western culture	20	65	20	15		80	20		50	45	5	
Only child					0.14			0.45				0.36
Yes	58	62	22	16		88	12		48	47	5	
No	84	45	33	21		83	17		42	46	12	
Child’s age					0.06			0.23				0.03
Under two years	45	67	20	13		80	20		60	31	9	
Two years or older	97	45	33	22		88	12		37	54	9	

* χ^2^-test.

**Table 5:** Distribution (%) of children’s and parents oral hygiene behaviour by the willingness to invest in children’s oral health.

		Willingness to Pay (Euro per Month)				Willingness to Visit the Dentist (Times per Year)			Willingness to Brush (Minutes per Day)			
	*n*	0–10 (%)	11–25 (%)	≥26 (%)	*p* *	0–2 (%)	≥3 (%)	*p* *	0–2 (%)	3–4 (%)	≥5 (%)	*p* *
Supervised brushing frequency					0.00			0.33				0.00
Once a day or less	68	68	21	12		88	12		56	43	1	
Twice a day or more	74	38	36	26		82	18		34	50	16	
Independent brushing frequency					0.81			0.49				0.43
Never	52	56	27	17		83	17		54	36	10	
Once a day or less	35	57	26	17		91	9		43	49	9	
Twice a day or more	55	46	33	22		84	16		36	56	9	
Age brushing was started ^†^					0.04			0.14				0.70
Before age one (*n* = 105)	105	57	29	14		84	16		43	47	10	
At age one or older (*n* = 33)	33	39	27	33		94	6		48	46	6	
In between meals sugar consumption					0.82			0.79				0.61
Four times a day or less	98	51	29	20		85	15		42	48	10	
More than four times a day	44	55	29	16		86	14		50	43	7	
Parents’ brushing frequency					0.01			0.06				0.10
Once a day or less	28	79	11	11		96	4		57	43	0	
Twice a day or more	114	46	33	21		82	18		41	47	11	
Parents’ in-between meals sugar consumption					0.63			0.62				0.99
Four times a day or less	68	52	26	22		87	13		44	47	9	
More than four times a day	74	53	31	16		84	16		45	46	9	

* χ^2^-test; ^†^ missing n = 4.

## Data Availability

The data presented in this study are available on reasonable request from the corresponding author. The data are not publicly available because the data are still needed for the Ph.D. project—it will be shared after completion of the Ph.D. project.
